# The relationship of neutrophil to lymphocyte ratio with testicular cancer

**DOI:** 10.1590/S1677-5538.IBJU.2019.0321

**Published:** 2020-01-13

**Authors:** Abdullah Ilktac, Bayram Dogan, Cevper Ersoz, Muzaffer Akcay, Habib Akbulut

**Affiliations:** 1 Department of Urology, Faculty of Medicine, Bezmialem Vakif University, Istanbul, Turkey

**Keywords:** Biomarkers, Orchiectomy, Testicular Neoplasms

## Abstract

**Purpose:**

To assess the relationship between testicular germ cell tumors (TGCT) and neutrophil to lymphocyte ratio (NLR) and to determine whether this ratio can be used as a serum tumor marker.

**Material and Methods:**

Sixty-one patients with testicular germ cell tumors were included into the study. Patients were grouped as localized and non-localized. Histologically patients were categorized as seminoma and nonseminomatous germ cell tumors. Complete blood cell count was measured the day before surgery and at the postoperative 1st month. Preoperative and postoperative mean NLR values were compared.

**Results:**

Thirty-six patients (59%) had seminomas and 25 patients (41%) had nonseminomatous testicular cancer. Forty-five patients (73.8%) had localized and 16 patients (26.2%) had non-localized testicular cancer. There was a statistically significant difference between preoperative and postoperative mean NLR of the localized patients (p=0.001) but no such difference was detected for non-localized patients (p=0.576). Nineteen patients with localized seminomas had normal preoperative serum tumor markers. There was a significant difference between preoperative and postoperative mean NLR in this group of patients (p=0.010). Twenty-six patients with localized tumors had preoperative increased serum tumor markers which normalized after orchiectomy. Mean NLR of these patients significantly decreased from 3.10±2.13 to 1.62±0.59 postoperatively (p=0.010).

**Conclusions:**

NLR appears to be a useful marker for TGCT. It is successful in predicting localized and non-localized disease in early postoperative period.

## INTRODUCTION

Testicular cancer is a relatively rare malignancy which forms 1% of all male cancers but it is the most common type of malignancy in men between ages of 15 and 44 (1, 2). Histologically, 90% of testicular cancers are germ cell tumors which divided into two groups as seminomatous and non-seminomatous germ cell tumors (3). Serum tumor markers play a crucial role in diagnosis, treatment and follow-up of patients with TGCT. There are three serum tumor markers currently used for TGCT: Alpha-fetoprotein (AFP), human chorionic gonadotropin (HCG) and lactate dehydrogenase (LDH). These markers are not very specific; AFP and HCG are increased in 50-70% and in 40-60% of patients with non-seminomatous germ cell tumors, respectively, whereas HCG elevation can be detected in only 30% of seminomas (4). LDH is a less specific marker reflecting growth rate and tumor volume, its level can be elevated in 80% of advanced seminomas and 60% of non-seminomatous tumors (5). In clinical stage 1 TGCT, fewer patients present with elevated serum tumor markers (6). Patients whose serum tumor markers normalize after orchiectomy are clinically disease free and surveillance or adjuvant treatment can be offered depending on the risk factors for occult metastatic disease (4). Patients with clinical stage 1 disease whose serum tumor markers remains elevated after orchiectomy are categorized as clinical stage 1S. These patients should be accepted to have systemic disease and treated with chemotherapy (7). Mostly TGCT metastasize via lymphatic route to retroperitoneal lymph node, lung, liver, bone and brain are other sites of metastasis (8).

There is increasing evidence supporting that inflammation plays a critical role in different aspects of cancer such as tumor development, progression and prognosis (9). Inflammatory cells produce several mediators and cytokines that can induce or promote angiogenesis, tumor growth, invasion and metastasis (10, 11). Also, it has been hypothesized that synthesis of inflammatory cytokines can be triggered by the tumor microenvironment resulting in alterations of acute phase reactants such as serum neutrophil and lymphocyte counts (12). There are several reports investigating relationship of various cancers with markers of systemic inflammatory response such as C-reactive protein, neutrophil count, platelet count and neutrophil to lymphocyte ratio (13). It has been reported that pretreatment NLR is related with recurrence and prognosis in colorectal, gastric, kidney and bladder cancers (14-16). NLR is defined as the absolute neutrophil count divided by absolute lymphocyte count and it is an inexpensive marker that can be easily acquired from complete blood cell parameters.

In this study, we aimed to reveal the relationship between TGCT and neutrophil to lymphocyte ratio and to determine whether this ratio can be used as a serum tumor marker for TGCT.

## MATERIAL AND METHODS

Data of the patients who underwent radical orchiectomy due to testicular cancer between 2014 and 2018 were analyzed retrospectively. Patients with testicular stromal tumors, infectious or inflammatory conditions, hematological disease, other malignancies, diabetes mellitus, cardiovascular diseases, end-stage renal disease, corticosteroid or B-agonist users and patients with missing data including preoperative and postoperative complete blood count, HCG, AFP, LDH and thoraco-abdominal tomography were excluded. Sixty-one patients with testicular germ cell tumor were included in the study whose preoperative and postoperative HCG, AFP, LDH and complete blood count values were available. Patients with no retroperitoneal or distant metastasis on computed tomography and no elevated serum markers following orchiectomy (stage 1A and stage 1B) were categorized as localized and patients with retroperitoneal or distant metastasis or elevated serum markers following orchiectomy (stage 1S, stage 2 and stage 3) were categorized as non-localized. Histologically patients were categorized as seminoma and non-seminomatous germ cell tumors.

Complete blood cell count was measured in the day before surgery and at the postoperative 1st month. NLR was defined as neutrophil count divided by lymphocyte count. Tumor markers were measured one week after surgery and for the patients whose marker levels declined but did not return to normal another measurement was performed 3 weeks later.

For statistical analysis, NCSS (Number Cruncher Statistical System) 2007 (Kaysville, Utah, USA) program was used. Study data were evaluated using descriptive statistical methods (mean, standard deviation, minimum, maximum, frequency and ratio). Mann Whitney U test was used for intergroup comparisons of quantitative data without normal distribution and Wilcoxon signed rank test was used for comparison of the changes seen in two paired measurements. Pearson's correlation coefficient was used to elucidate the correlation between tumor size and preoperative NLR. A p value <0.05 was considered statistically significant. Receiver operating characteristics curve analysis was performed to find cut-off levels for NLR as a predictor of localized and non-localized TGCT.

## RESULTS

**Figure 1:**
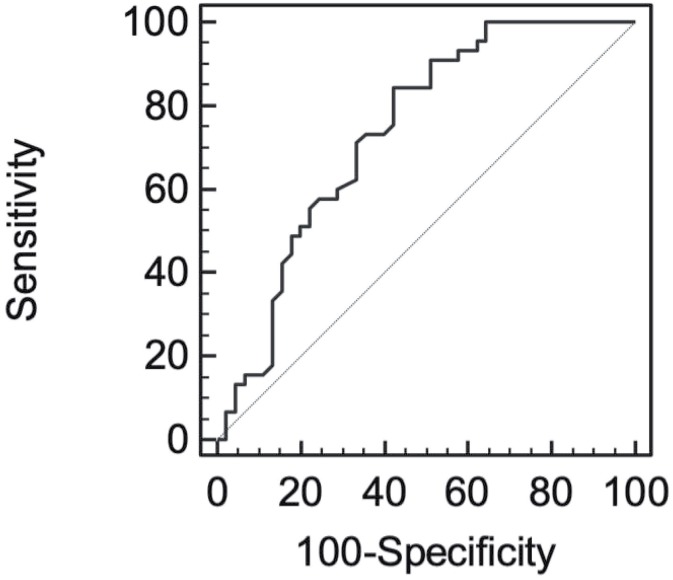
Receiver operating curve used to define optimal cut off value of NLR for localized TGCT.

**Figure 2:**
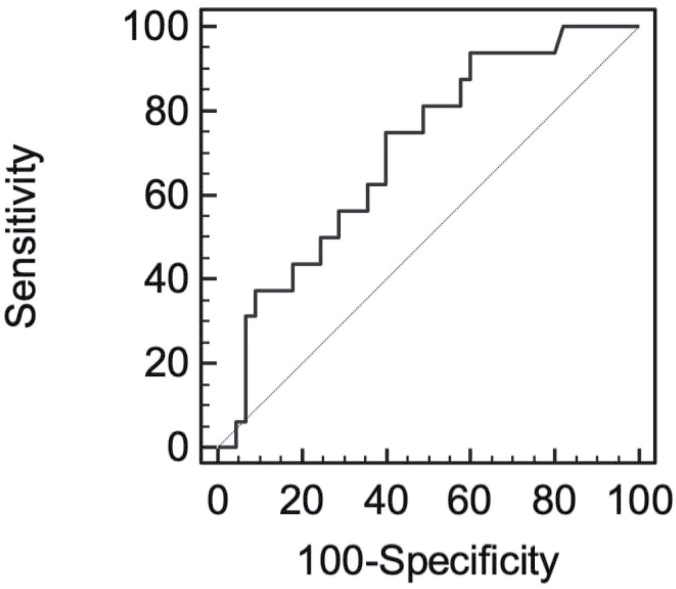
Receiver operating curve used to define optimal cut off value of NLR for non-localized TGCT.

Mean age of the patients was 37.83±9.98 years. Thirty-six patients (59%) had seminomas and 25 patients (41%) had nonseminomatous testicular cancer. Forty-five patients (73.8%) had localized and 16 patients (26.2%) had non-localized testicular cancer. Demographic characteristics of the patients are summarized in Table-1. All of the nonseminomatous tumors were mixed germ cell tumors; 6 patients had embryonal carcinoma and yolk sac tumor, 9 patients had embryonal carcinoma, yolk sac tumor and teratoma, 5 patients had teratoma and embryonal carcinoma, 4 patients had teratoma and yolk sac tumor and 1 patient had choriocarcinoma, yolk sac tumor and teratoma. There was a statistically significant difference between preoperative and postoperative mean NLR of the localized patients (preoperative NLR: 2.78±1.84, postoperative NLR: 1.57±0.58, p=0.001) but there was no statistically significant difference between preoperative and postoperative mean NLR of non-localized patients (preoperative NLR: 3.83±1.65, postoperative NLR: 3.52±2.79, p=0.576) (Table 2). Mean preoperative and postoperative NLR was significantly higher in non-localized patients compared to localized patients (Table-3). Thirteen seminomatous and 13 non-seminomatous, a total of 26 patients with localized TGCT had preoperative increased serum tumor markers (elevated HCG and/or AFP and/or LDH) which normalized after orchiectomy. Mean NLR of these patients significantly decreased from 3.10±2.13 to 1.62±0.59 postoperatively (p=0.010). Nineteen patients had normal preoperative serum tumor markers and all of these patients had localized seminomatous TGCT. Preoperative mean NLR of these patients was 2.43±1.28 and this ratio decreased to 1.55±0.57 postoperatively. This difference was also statistically significant (p=0.010) (Table-4). Pearson's correlation coefficient was calculated as 0.302 indicating a positive correlation between tumor size and preoperative NLR that was statistically significant (p=0.018). Preoperative and postoperative NLR of localized TGCT were used to define an optimal cut off value for the presence of localized TGCT. The optimal cut off value for localized TGCT was 2.11 with sensitivity 84.44% and specificity 57.78%, area under the receiver operating characteristics curve was 0.73 (95% confidence interval=70.5-93.5) (Figure-1). Preoperative NLR of localized and non-localized TGCT were used to define an optimal cut-off value for the presence of non-localized TGCT. The optimal cut off value for non-localized TGCT was 2.56 with sensitivity 75% and specificity 60%, area under the receiver operating characteristics curve was 0.703 (95% confidence interval=47.6-92.2) (Figure-2).

**Table 1:** Demographic characteristics of the patients.

Age, years, mean±SD (range)	37.83±9.98	(20-65)
Tumor size, cm, mean±SD (range)	4.45±1.93	(0.4-12)
Total number of patients, n (%)	61	(100)
Localized	45	(73.8)
Non-localized	16	(26.2)
Seminoma	36	(59)
Non-seminoma	25	(41)

**Table 2:** Preoperative and postoperative neutrophil to lymphocyte ratio in localized and non-localized TGCT.

	Preop. NLR	Postop. NLR	P
Localized TGCT (mean±SD)	2.78±1.84	1.57±0.58	0.001
Non-localized TGCT (mean±SD)	3.83±1.65	3.52±2.79	0.576

**TGCT =** Testicular germ cell tumor; **Preop =** Preoperative; **Postop =** Postoperative **NLR =** Neutrophil to lymphocyte ratio

**Table 3:** Comparison of neutrophil to lymphocyte ratio between localized and non-localized TGCT.

	Localized	Non-localized	P
Preop. NLR (mean±SD)	2.78±1.84	3.83±1.65	0.016
Postop. NLR (mean±SD)	1.57±0.58	3.52±2.79	0.004

**TGCT =** Testicular germ cell tumor; **Preop =** Preoperative; **Postop =** Postoperative; **NLR =** Neutrophil to lymphocyte ratio.

**Table 4:** Neutrophil to lymphocyte ratio of patients with preoperative elevated tumor markers that normalized postoperatively and patients with normal tumor markers.

	Preop. NLR	Postop. NLR	P
Preop. elevated markers (mean±SD)	3.10±2.13	1.62±0.59	0.010
Marker normal patients (mean±SD)	2.43±1.28	1.55±0.57	0.010

**Preop =** Preoperative; **Postop =** Postoperative; **NLR =** Neutrophil to lymphocyte ratio

## DISCUSSION

Although testicular cancer is the most common cancer in men under 40 years of age, it is curable in most of the cases (17). Germ cell tumors constitute 90% of all testicular cancers and serum tumor markers play a crucial rule in diagnosis, treatment and follow-up of patients with TGCT (3). NLR is an inexpensive and easily acquired inflammatory marker. Association of NLR and cancer was widely studied in various malignancies (14, 15) but the number of studies investigating the relationship between NLR and testicular cancer is quite low. Yuksel et al. compared preoperative NLR of patients with localized TGCT with varicocele patients who were included in the study as control group and found that NLR was significantly higher in patients with testicular cancer (18). In our study, we included both localized and non-localized patients and compared preoperative NLR with postoperative NLR. In localized patients there was a significant decrease in NLR after orchiectomy. Whereas, in non-localized patients there was not any statistically significant change in NLR value after surgery. It is known that patients with localized TGCT are mostly cured after orchiectomy whereas in non-localized patients there is still residual tumor. Maintaining an elevated NLR after orchiectomy is related with non-localized testicular cancer and observing a significant decrease in postoperative NLR suggests that patient has a localized disease. There is no other study in literature evaluating preoperative and postoperative NLR in testicular cancer. Park et al. investigated pretreatment and post-treatment NLR in patients with metastatic renal cell carcinoma receiving sunitinib (19). Post-treatment NLR was measured after first cycle of sunitinib treatment and they found that better tumor response was significantly associated with lower post-treatment NLR and larger reduction in NLR after first cycle. Morizawa et al. evaluated pretreatment and post-treatment NLR in patients with muscle invasive bladder cancer who underwent radical cystectomy (20). They stated that preoperative NLR decreased postoperatively and remained low in non-recurrent cases during follow-up. In recurrent cases although NLR decreased temporarily after radical cystectomy it increased significantly before recurrence was detected on imaging methods.

When mean preoperative NLR of localized patients was compared with non-localized patients, it was seen that NLR of non-localized TGCT patients was significantly higher. This significance was even higher when we compared mean postoperative NLR of these two groups as expected. This data suggest that as the disease progresses, there is an increase in NLR also. As far as we know there is not any other study in literature investigating the relationship of NLR with localized and non-localized TGCT. NLR has been showed to be an independent risk factor in various other urological tumors such as renal cancer, bladder cancer, upper tract urothelial carcinoma and prostate cancer (21). There is only one study regarding the predictive value of NLR on the prognosis of TGCT (13). Fifty-three patients with germ cell tumor were evaluated and no relationship was found between preoperative NLR and cancer specific survival or progression free survival.

AFP, HCG and LDH are important tumor markers that are helpful in diagnosis, staging and evaluation of response to the therapy (6). The persistence of elevated serum tumor markers after orchiectomy might indicate the presence of metastatic disease (macro-or microscopically), while the normalization of marker levels after orchiectomy does not rule out the presence of tumor metastases (4). In our study, twenty-six patients with localized TGCT had preoperative increased serum tumor markers (elevated HCG and/or AFP and/or LDH) that normalized postoperatively. Mean NLR of these 26 patients significantly decreased after orchiectomy. This finding shows us that there is a correlation between NLR and conventional serum tumor markers. Nineteen patients had normal serum markers preoperatively. All of these patients had localized seminomatous TGCT. When we compared preoperative NLR with postoperative NLR of this group we found that there is a significant decrease indicating that in seminomas with normal pre-orchiectomy serum markers, NLR can be used as an alternative marker.

Recently microRNAs, small noncoding RNAs involved in epigenetic regulation of gene expression, have been suggested as novel biomarkers for TGCT. Among these, microRNA-371a-3p has been proven to be the most promising. Dieckmann et al. found that microRNA-371a-3p has a sensitivity of 90%, specificity of 94% and positive predictive value of 97% for primary diagnosis of TGCT (22). It was also shown that its plasma levels began to decrease within hours after orchiectomy in patients with localized TGCT, remained elevated in patients with non-localized disease and also levels dropped after treatment were found to be elevated with relapse (22-24). These results suggest that microRNA-371a-3p is a more useful marker for TGCT than both conventional markers and NLR. MicroRNA-371a-3p has not yet received regulatory approval but it is expected to be implemented in routine clinical practice soon (25).

We performed receiver operating characteristics curve analysis to define cut-off levels for NLR as a predictor of localized and non-localized TGCT. The optimal cut off value for localized TGCT was 2.11. The optimal cut off value for non-localized TGCT was 2.56. In their study, Yuksel et al. defined a cut off value of 2.06 for NLR in localized TGCT similar to our finding (18). Bolat et al. defined optimal cut off value of NLR for progression free survival as 3.55 and for cancer specific survival as 3.0 (13).

## CONCLUSIONS

NLR appears to be a useful marker for predicting localized and non-localized TGCT in early postoperative period. A significant decrease in NLR after orchiectomy, especially a value of less than 2, indicates localized disease. Absence of a significant reduction after orchiectomy, especially a value greater than 2.5 indicates non-localized disease. In patients who have seminomas with normal conventional serum markers, NLR can be used as an alternative marker to differentiate localized disease. Our study has several limitations. We had a small group of patients and we did not evaluate relationship of NLR with recurrence and prognosis. According to our results NLR seems to be inferior to microRNA-371a-3p as a marker for TGCT. Also, we measured NLR one month after surgery. We didn't have any early postoperative measurement so we could not simultaneously compare NLR with conventional markers and we could not determine the half-life of NLR. Further large-scale and prospective studies are required to support our results. Also, it would be very valuable if it can be proven that NLR normalized after orchiectomy significantly increases with recurrence.
